# Should ChatGPT be considered a medical writer?

**DOI:** 10.51866/lte.483

**Published:** 2023-11-28

**Authors:** Apichai Wattanapisit, Apichat Photia, Sanhapan Wattanapisit

**Affiliations:** 1 MD, Dip., Thai Board of Family Medicine, Academic Fellowship Department of Clinical Medicine, School of Medicine, Walailak University, Thasala, Nakhon Si Thammarat, Thailand.; 2 Family Medicine Clinic, Walailak University Hospital, Nakhon Si Thammarat, Thailand. Email: apichai.wa@wu.ac.th; 3 MD, Dip., Thai Board of Pediatrics, Pediatric Hematology and Oncology, Research Fellowship Phramongkutklao Hospital and College of Medicine, Bangkok, Thailand.; 4 MD, Dip., Thai Board of Family Medicine, MSc Family Medicine Unit, Thasala Hospital, Nakhon Si Thammarat, Thailand.

**Keywords:** Authorship, ChatGPT, Writing

## Dear editor,

The recent commentary titled ‘Use of ChatGPT in medical research and scientific writing’ by Lee et al. provides insights into the use of ChatGPT in research and writing, including potential risks.^1^ We agree that ChatGPT cannot completely replace human work and does not meet the authorship criteria. In this letter, we discuss perspectives about our experience.

The use of ChatGPT, an artificial intelligence (AI) chatbot, for medical writing has been widely debated. On 20 April 2023, we conducted a search using the term ‘ChatGPT’ in PubMed and found 282 results. On 30 September 2023, another search yielded 1336 results, indicating a massive increase in the number of publications related to ChatGPT. The AI chatbot can generate texts for a scientific article, such as an abstract or a manuscript. The generative pretrained transformer, an Al-based language model, has been employed as a helpful function in some articles, leading to ChatGPT being considered a ‘co-author’. On 20 April 2023, an advanced PubMed search was also conducted using the term ‘ChatGPT[Author] ’, which identified an article. However, a corrigendum to the aforementioned article was published to remove ChatGPT from the author list owing to failure of meeting the authorship criteria.^2,3^ In particular, the AI chatbot failed to meet the following criterion: Each author must ‘be accountable for all aspects of the work’.^4^

In our experience, ChatGPT (GPT-3.5) (OpenAI, CA, USA) could generate a prompt paragraph within a few seconds. We typed ‘write an introduction for a scholarly manuscript: [proposed title of the manuscript]’, and the AI chatbot generated a 139-word paragraph relevant to the topic. Subsequently, we asked the AI chatbot to ‘expand this into three short paragraphs with references’. The prompt yielded 282 words in three paragraphs with four references. The contents and quality of the writing were satisfactory. We also found the references cited in the AI-generated paragraphs and a complete reference list at the end of the texts. We checked the references by searching the article titles in PubMed (all four journals indexed in PubMed) and the journal websites. However, we did not find any cited articles. In other words, those articles do not exist in the literature.

In summary, our experiment testing ChatGPT’s ability to generate a scholarly manuscript introduction and subsequent expansion with references yielded satisfactory results. However, we discovered that all references provided by the AI chatbot were non-existent, highlighting the unreliability of relying solely on ChatGPT as a medical writer. This issue challenges the human intellect and ethical considerations in medical writing. Therefore, it is crucial to consider using ChatGPT as a tool for guidance rather than a replacement for human expertise. While ChatGPT’s language generation capabilities are impressive, it is essential to exercise caution and verification when using AI for medical writing purposes.
